# Implications for conservation and game management of the roadkill levels of the endemic Iberian hare (*Lepus granatensis*)

**DOI:** 10.1038/s41598-021-00147-3

**Published:** 2021-10-19

**Authors:** Jesús Duarte, David Romero, Pablo J. Rubio, Miguel A. Farfán, Julia E. Fa

**Affiliations:** 1 Ofitecma Marbella, Av. Ramón y Cajal 17, 29601 Marbella, Spain; 2 Biogeography, Diversity and Conservation Research Group, Department of Animal Biology, Faculty of Sciences, University of Málaga, 29071 Málaga, Spain; 3 Delegación de Medio Ambiente, Oficina Técnica, Mancomunidad de Municipios de la Costa del Sol Occidental, Calle de Bonanza s/n, 29604 Marbella, Spain; 4 Division of Biology and Conservation Ecology, Manchester Metropolitan University, Manchester, UK

**Keywords:** Ecology, Zoology, Environmental sciences

## Abstract

The Iberian hare (*Lepus granatensis*) is an important small game species endemic to the Iberian Peninsula for which the incidence of roadkill is unknown. We surveyed Iberian hare–vehicle accidents on road networks in southern Spain, focusing on roads that mainly run through favorable habitats for this species: Mediterranean landscapes with plots of arable crops, olive groves, and vineyards. We recorded roadkills over a 5-month period, estimated hare accident densities on roads, and compared these numbers to hare hunting yields in adjoining hunting estates. We also analyzed the spatial patterns of and potential factors influencing hare roadkills. We detected the existence of black spots for hare roadkills in areas with high landscape heterogeneity that also included embankments and nearby crossroads and had high traffic intensity. Hare roadkill levels ranged from 5 to 25% of the annual harvest of hares killed on neighboring hunting estates. We suggest that road collisions should be considered in Iberian hare conservation in addition to hunting, since they may represent an additive source of mortality. Game managers should address the issue of hare roadkill in harvest planning to compensate for hare accidents, adjusting hunting quotas to account for this unnatural source of mortality. Our results suggest future directions for applied research in road ecology, including further work on demographic compensation and roadkill mitigation.

## Introduction

Roads impact wildlife populations through landscape fragmentation, loss of connectivity, emergence of corridors favoring anthropogenic species or predators, and direct mortality^1^. Further, roads have been identified as one of the main threats to the conservation of mammals globally^2^. Roads can cause declines in the carrying capacity of a species either directly through habitat destruction or through the indirect modification of areas of up to 100 m on either side of the road^3^. Human activity and traffic noise associated with roads can also disturb adjacent habitats^4^. Roads can impair biological activities that require movement, such as reproduction, feeding, or dispersal, resulting in genetic isolation^5,6^ and affecting the demography, spatial distribution, and abundance of species^7,8^.

Many species are affected by road collisions, from large vertebrates and mesocarnivores^9–11^ to smaller animals^12–15^. However, the incidence of road mortality in lagomorphs has not been well studied. Previous research examining the impacts of roads on hares has focused on the European hare (*Lepus europaeus*)^8,16,17^ and other non-Mediterranean hare species^18–20^. To date, such data has not been gathered on the Iberian hare (*Lepus granatensis*), nor has a detailed analysis of road mortality been conducted for this species.

The Iberian hare is endemic to the Iberian Peninsula^21^. These hares are medium-sized, largely nocturnal lagomorphs that inhabit pastureland, farmland, plains, and forests throughout their range, as well as mountainous scrubland areas in the northern part of their distribution^22^. Although hares are important game animals^23^, little is known about their population biology and demography (but see^24–27^). Specifically, few studies have quantified non-hunting mortality rates, and even fewer have addressed the additive effect of hunting and roadkill on this game species. Between 13 and 38% of the hare populations studied are known to be affected by predation, disease, and environmental events (e.g., floods)^28,29^. Although it is known that road mortality has adverse effects on wildlife^6,13^, few studies have specifically addressed Iberian hare roadkills. It has been suggested that in the northern part of its range in Spain, only a small portion of Iberian hare mortality (9%) is due to roadkill^29^. However, road mortality is likely more frequent in southern Spain, where large areas are dedicated to the growing of cereals, sunflowers, grapes, and olives^30^, providing favorable habitat for this species.

Road collisions may serve as a significant source of mortality for some game species, such as hares^17^, so the ratio of collisions to annual harvest numbers should be considered in the management of populations with high road mortality. In Andalusia (a region in southern Spain), the average annual hare harvest ranges from 0.8 to 20.9 hares/km^2^^31^. Nearly 250,000 hares are hunted per year^32^, which clearly demonstrates the economic importance of this species. It is therefore vital to understand the ecological factors that influence mortality in Iberian hare populations, given the taxonomic importance of this endemic species and the significant role it plays in the ecology and rural economy of the region. Demographic compensation is a frequent response in short-lived species^33^ such as the Iberian hare. Therefore, road collisions, as a type of additive mortality, must be considered in management or hunting plans for this species.

Identifying relationships between hare roadkill patterns and characteristics of the landscape is essential to propose collision mitigation measures for conservation purposes. This study assessed Iberian hare roadkill within a large area of their distribution in southern Spain for which there is hardly any data on road mortality. We hypothesized that, apart from hunting activities, road mortality may be a significant cause of death for this species and thus may affect its population ecology and condition its hunting exploitation. Our objectives were to quantify roadkill rates and compare them to the harvest rates recorded on neighboring hunting estates. We also identified black spots with high roadkill rates and the factors likely to be associated with these. Finally, we propose management measures for the conservation of these populations, which we hope will also be applicable to other regions and spatial scales. Thus, if the additive mortality of Iberian hares due to road collisions can lead to overhunting, this could also occur for other game species or species of ecological interest, affecting their population dynamics such that it may be advisable to adjust their hunting quotas.

## Material and methods

### Study area

The study was conducted in Antequera County (37° 10′ N, 4° 37′ W) in northeast Malaga province (Andalusia, southern Spain) during 2006. The study area comprised seven municipalities with an approximate total area of 1269.9 km^2^. The asphalt road network within these municipalities was 391.8 km in length, not including highways^34^. The area is characterized by a continental Mediterranean climate, with mean temperatures ranging from 26 °C in August to 9 °C in January. Annual rainfall is 550 mm and is concentrated between October and May. Summers are dry and hot, and winters are cold; days with snow are rare, although frost may occur in winter since evening temperatures may fall below − 3 °C^35^.

The area is a relatively flat (400–550 m elevation) fertile plain, over 80% of which is covered by farmland. Typical crops include olives, grapes, sunflowers, cereals, and other dry herbaceous plants. Natural vegetation is concentrated along the adjoining hills or in small habitat islands within or between crop fields. These are dominated by scattered holm oak (*Quercus rotundifolia*), wild olives (*Olea europaea* var. *sylvestris*), and dense scrubland consisting of rockroses (*Cistus* spp.), mastic trees (*Pistacia lentiscus*), and various Labiatae^36^. Other types of natural vegetation are present along hedges, crop boundaries, and road borders, where the plant community is dominated by annual herbaceous and nitrophilous species^37^.

Densities of Iberian hares are highly variable (between 15 and 33 hares/km^2^) depending on habitat and environmental conditions^38^. In Andalusia, exceptionally high densities of over 100 hares/km^2^ are reached in Doñana National Park in years when flooded area and herbaceous cover increase. In the study area, density ranges between 50 and 80 hares/km^2^^39^, which is higher than the average value in Andalusia. Olive groves combined with other crops may be a favorable habitat for this species^23,40^, as they provide habitat heterogeneity, vegetative cover, food, and water (in the case of drip irrigation of olive trees).

### Data collection

**Figure 1:**
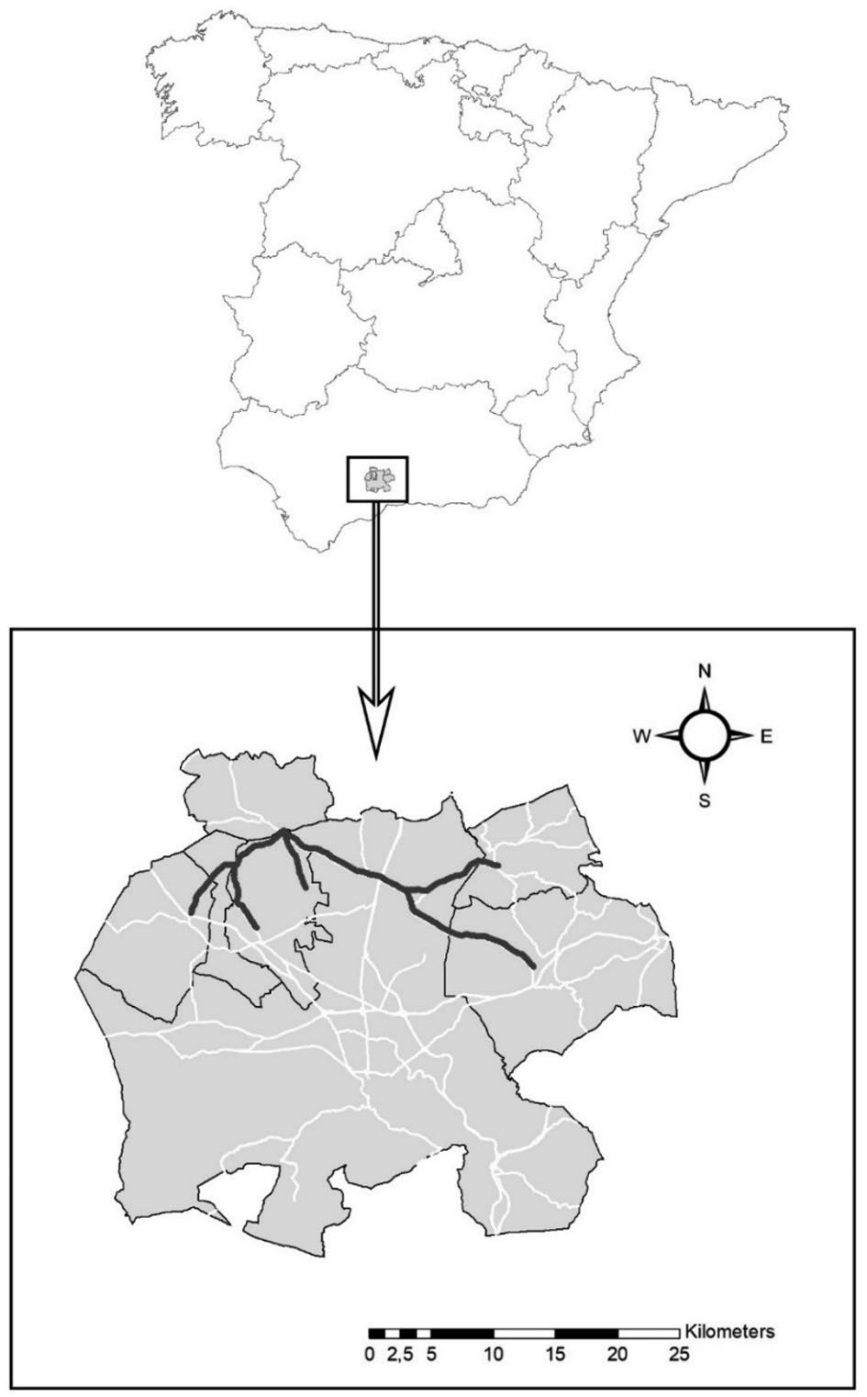
Location of the study area in northeast Malaga province (southern Spain), showing the stretches of road sampled for hare roadkills (thick black lines; 55.7 km) in relation to the total road network in the study area (white lines). Map created with ArcGis 9.3 (ESRI 2008). Datasets from Instituto de Estadística y Cartografía de Andalucía, Junta de Andalucía. Road network and administrative boundaries (https://www.ideandalucia.es/catalogo/inspire/srv/spa/catalog.search#/home).

We selected seven main roads within the study area on which to perform roadkill counts (Fig. 1, Table 1). The sampled area comprised a rectangle of 30 × 15 km, which included a total of 55.7 km of roads (14.2% of the total road network). All roads were composed of two lanes (one running in each direction), with an asphalt surface 6–7 m wide and a 1–2 m shoulder on either side. Verges with vegetation were present between the roads and the surrounding cropland. The maximum speed on all the selected roads was 90–100 km/h, although some sections had lower speed limits. The mean traffic intensity for 2005 was nearly 1000 vehicles/day, ranging from 500 to 2000 vehicles^35^. However, the traffic intensity has increased up to 5000 vehicles/day on two of the analyzed roads in the last 10 years^41^. All roads were unfenced, allowing wildlife to cross and access the surrounding vegetation. We excluded highways from our analysis because these were all fenced and therefore exhibited an absence of roadkills during prior sampling.

**Table 1:** Features of the roads sampled in the study area.

Road code	Name	Length	Traffic volume
MA-5101	Archidona—Villanueva de Algaidas	12.7	500
MA-6414	Villanueva de Algaidas—Córdoba	11.8	1000–2000
MA-6415	Córdoba—Alameda	8.9	1000–2000
MA-6409	Alameda—Los Carvajales	5.1	500–1000
MA-6410	Los Carvajales—Mollina	5.8	500–1000
MA-6408	Los Carvajales—Fuente de Piedra	5.7	500–1000
S/C	Alameda—Cortijo Peinado	5.7	500
	Total	55.7	

Road length is given in kilometers. Traffic volume represents the average number of vehicles/day estimated on the road^34,41^.

Roads were surveyed weekly for 5 months, between March 1, 2006, and July 31, 2006. This is the period of maximum reproductive activity for hares in the region^26^. Before the start of the sampling period, we removed all carcasses from the selected road sections. Surveys were carried out at dawn and were conducted from a car driven at 10 km/h, as surveying the roads by foot was prohibited by the police. Three surveyors were present during each survey, and surveyors remained the same throughout the study period to avoid inter-observer biases. When an *L. granatensis* carcass was detected, we recorded the UTM coordinates of the collision point using a GPS eTrex Vista Cx (Garmin, USA) and then removed the carcass to avoid double counting during subsequent sampling. All other wild species killed by vehicle collisions were also recorded. Kill rates were standardized as the number recorded per 100 km^19^.

We rely on the concept of road effect zone (REZ)^42^, i.e., the impact of a road on its surrounding area and the distance to which the road disturbs habitat and wildlife, to investigate whether hare roadkills were aggregated in certain road sections (e.g., black spots) and to estimate the density of roadkills within buffer zones of a certain size. REZ varies greatly depending on roadside features (upslope or downslope), traffic intensity, type of road (primary or secondary), and the part of the ecosystem affected, among other factors^43,44^. For rural secondary roads with traffic intensities exceeding 10,000 vehicles/day, the REZ is greater than 200 m^44^. The impact of roads on herbaceous species, the main habitat resource for hares, reaches up to 100 m^42^, whereas the impact on wildlife distribution may reach from 250 m for small species up to 500–1000 m for larger wildlife such as ungulates^45,46^. Therefore, we considered two conservative approaches for estimating REZ for the Iberian hare: 100 m and 500 m, the first being an estimate of the possible impact of roads on its habitat resources and the second being the maximum distance to which the road might affect a medium-sized species. Accordingly, we identified possible black spots along 100 and 500 m road sections and computed the density of roadkills (number of hares killed per km^2^) in buffer areas of the same radii.

To compare the number of hares killed on hunting estates with the number killed on roads, we used the annual hunting reports (AHRs) from 181 game estates for the period 1993–2001. These comprise all the game estates in the seven selected municipalities that were traversed by the sampled roads. We analyzed 1282 AHRs from these estates and estimated the hunting yield (HY) as ∑ mean annual number of hares hunted per game estate/∑ areas of the game estates in km^2^^47–49^. Hunting data were taken from previous studies in the area^32^. As our estimation of hare roadkill densities was based on a study period of 5 months while the hare hunting season in Andalusia lasts for only 3 months, we standardized densities and yields as the mean rate per month and compared them by calculating a ratio of accident densities to hunting yields.

### Roadkill modeling

The number of collisions for any given species depends on several factors related to road features and traffic volume^50,51^ as well as animal behavior and phenology^7,52,53^. The structure of the surrounding habitat and landscape can also play an important role^1,14,54^. To determine the importance of these possible factors in our study, we overlaid hare collision points on habitat maps derived from digital validated and institutional orthophotographs (scale 0.5 m/pixel)^55^ using ArcGIS 9.3 software (Esri, USA). All roads containing collision points were also digitized onto the habitat maps. During the hare surveys, we also recorded the environmental characteristics and land uses of the surrounding landscape to validate the data from the orthophotographs.

We quantified variables related to the road, surrounding habitat, and landscape (Table 2) at each collision point, considering two sampling levels (in accordance with the two ERZ approaches): a buffer of 100 m radius around each collision point for the habitat level (see^56,57^) and another buffer of 500 m radius for the landscape level^58^. At the habitat level, we noted the various crops and types of natural vegetation present and measured the surface area of each vegetation patch. We also estimated habitat diversity using the Shannon index^59^. At the landscape level, we measured the length of the ecotone and estimated land heterogeneity using the Baxter-Wolfe interspersion index^60,61^ along a transect perpendicular to the road.

**Table 2:** Variables measured to model the factors affecting hare–vehicle collision locations.

Code	Definition
**Road features**	
Traffic (Tf)	Estimated traffic volume (vehicles/day; classes: 1. < 500; 2. 500–1000; 3. 1000–2000)
Speed limit (Sl)	Road section speed limit (km/h)
Cross (Dc)	Distance to nearest crossroad (m)
Embankment (Em)	Presence of embankment (road above surrounding land) (P/A)
Slope (Sp)	Presence of lateral cutting (road below surrounding land) (P/A)
Ditch (Dt)	Presence of marginal ditch (P/A)
**Habitat features**	
Crops (Cp)	Total surface area covered by crops (ha)
Natural (Nv)	Total surface area covered by natural vegetation (ha)
Diversity (Pd)	Patch diversity (Shannon index; crops and natural vegetation)
**Landscape features**	
Ecotone (Ec)	Total ecotone length (km)
Heterogeneity (Lh)	Landscape heterogeneity (Baxter-Wolfe interspersion index)

Road features were measured at each collision point. Habitat-level variables were measured in a 100 m radius buffer around each collision point, while landscape-level variables were measured in a 500 m radius buffer.

*P/A* presence/absence.

We also generated 81 control points, separated by 500 m and in areas where no roadkills were detected, to compare the environmental conditions between points with and without roadkill. We applied the same procedures as used for the collision points regarding the buffers and environmental variable measurements.

### Data analysis

We used the Kolmogorov–Smirnov test to examine whether temporal (weekly) roadkill numbers followed a uniform or random (Poisson distribution) pattern and tested whether the spatial pattern of collisions in each road section fit a pattern expected at random through the Wald-Wolfowitz run test^62^. If the random hypothesis was rejected, we estimated a spatial index of dispersion as the variance/mean ratio (VMR). If this ratio yielded values > 1, hares roadkills were considered to be dispersed as contagious objects^63^ in those road sections.

To test for multicollinearity between variables, we developed a correlation matrix and obtained the Spearman’s rank correlation coefficient (rho). Based on this value, the coefficient of determination (R^2^) and variance inflation factor (VIF) were calculated to measure collinearity between variables (VIF > 5^64^), removing one of the variables involved in the cases. Only those that captured the effects of any set of highly correlated variables could continue. The VIF was calculated as:

$$ {\text{VIFi}} = {1 \mathord{\left/ {\vphantom {1 {\left( {1 - {\text{R}}^{2} {\text{i}}} \right)}}} \right. \kern-\nulldelimiterspace} {\left( {1 - {\text{R}}^{2} {\text{i}}} \right)}}. $$

We generated predictive models for hare roadkills using generalized linear mixed-effects models (GLMMs) with a binomial error distribution and logit link function^65^ to test whether the probability of detecting a hare collision was related to any of the road or environmental factors. The presence/absence of roadkill was used as the dependent variable (collision point = 1, random point without collisions = 0), while the different roads sampled were incorporated as a random factor. We compared models using Akaike’s Information Criterion corrected for small samples (AICc)^66^ and selected the model with the lowest AICc. Statistical analyses were conducted using the SPSS 24.0 software package (IBM, USA). Means are presented with their standard errors.

### Updating the surveys

As 15 years have elapsed since the surveys in this study were conducted, both road conditions and hare densities or hunting yields may have changed. Therefore, we conducted additional surveys to examine whether the current road conditions, roadkill densities and hunting yields were similar to those from the initial data collection period.

Using the most updated data available, we checked for differences between mean traffic volume in the roads sampled. We used the traffic data from 2017 to compare. Secondly, we carried out supplementary roadkill surveys for 4 weeks during June–July 2021 over the same roads that were sampled in 2006. Finally, we also reviewed the hare hunting yields in Málaga for 2019 and carried out interviews with game managers (N = 10 game estates) in the area to obtain their hare hunting yields from the last season.

## Results

A total of 1336.8 km of roads were sampled during the 5-month study period, involving 171.9 observer hours. We recorded a total of 162 carcasses: 68.5% Iberian hares, 17.9% wild rabbits, 4.9% other mammals, 5.6% birds, and 3.1% reptiles (Table 3). Of the 111 Iberian hares detected, only 80 of the deaths could be clearly attributed to a vehicle collision; these were considered for further spatial analysis.

**Table 3:** Species detected during sampling for animal–vehicle accidents in the study area (March–July 2006).

Species	n	%
**Mammals**		
Iberian hare (*Lepus granatensis*)	111	68.52
Wild rabbit (*Oryctolagus cuniculus*)	29	17.90
Rodents (*Rattus* sp.)	2	1.23
Western hedgehog (*Erinaceus europaeus*)	2	1.23
Red fox (*Vulpes vulpes*)	2	1.23
Common genet (*Genetta genetta*)	1	0.62
Western polecat (*Mustela putorius*)	1	0.62
**Birds**		
Little owl (*Athene noctua*)	6	3.70
Short-toed eagle (*Circaetus gallicus*)	1	0.62
Red-necked nightjar (*Caprimulgus ruficollis*)	1	0.62
Mallard (*Anas platyrhynchos*)	1	0.62
**Reptiles**		
Montpellier snake (*Malpolon monspessulanus*)	3	1.85
Other snakes	2	1.23
Total	162	

**Figure 2:**
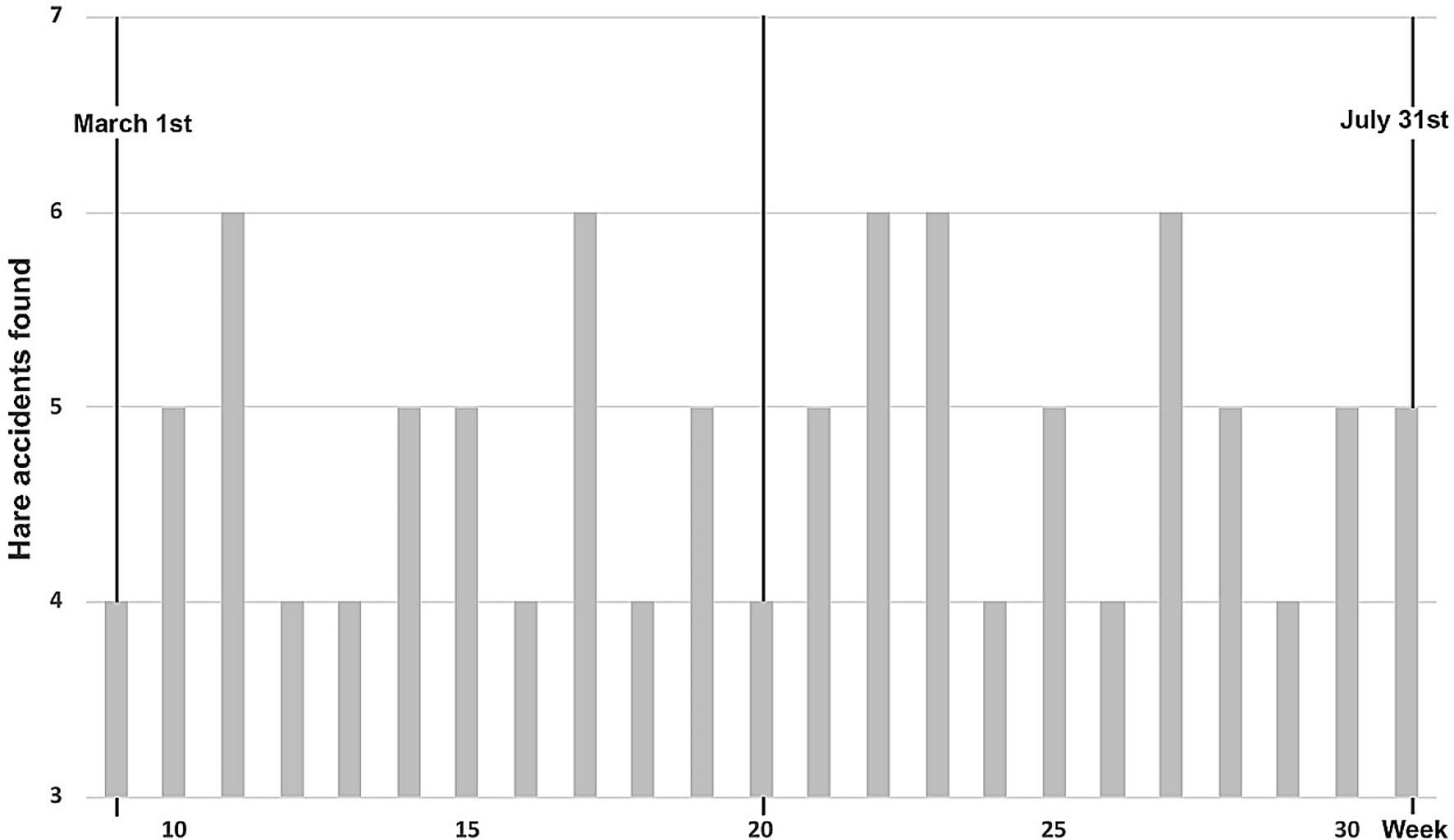
Weekly Iberian hare accidents during the study period.

An average of 4.8 ± 0.2 hare accidents were detected per week, with 4–6 hares found dead weekly on the sampled roads (Fig. 2). From a temporal point of view, hare roadkills significantly differ from a uniform weekly pattern (Kolmogorov–Smirnov: N = 23; weeks; Z = 1.877; p = 0.002) and from a random pattern (Kolmogorov–Smirnov: N = 23; Z = 1.392; p = 0.041). We estimated a standardized kill rate of 7.2 hares/100 km per week in the study area.

**Figure 3:**
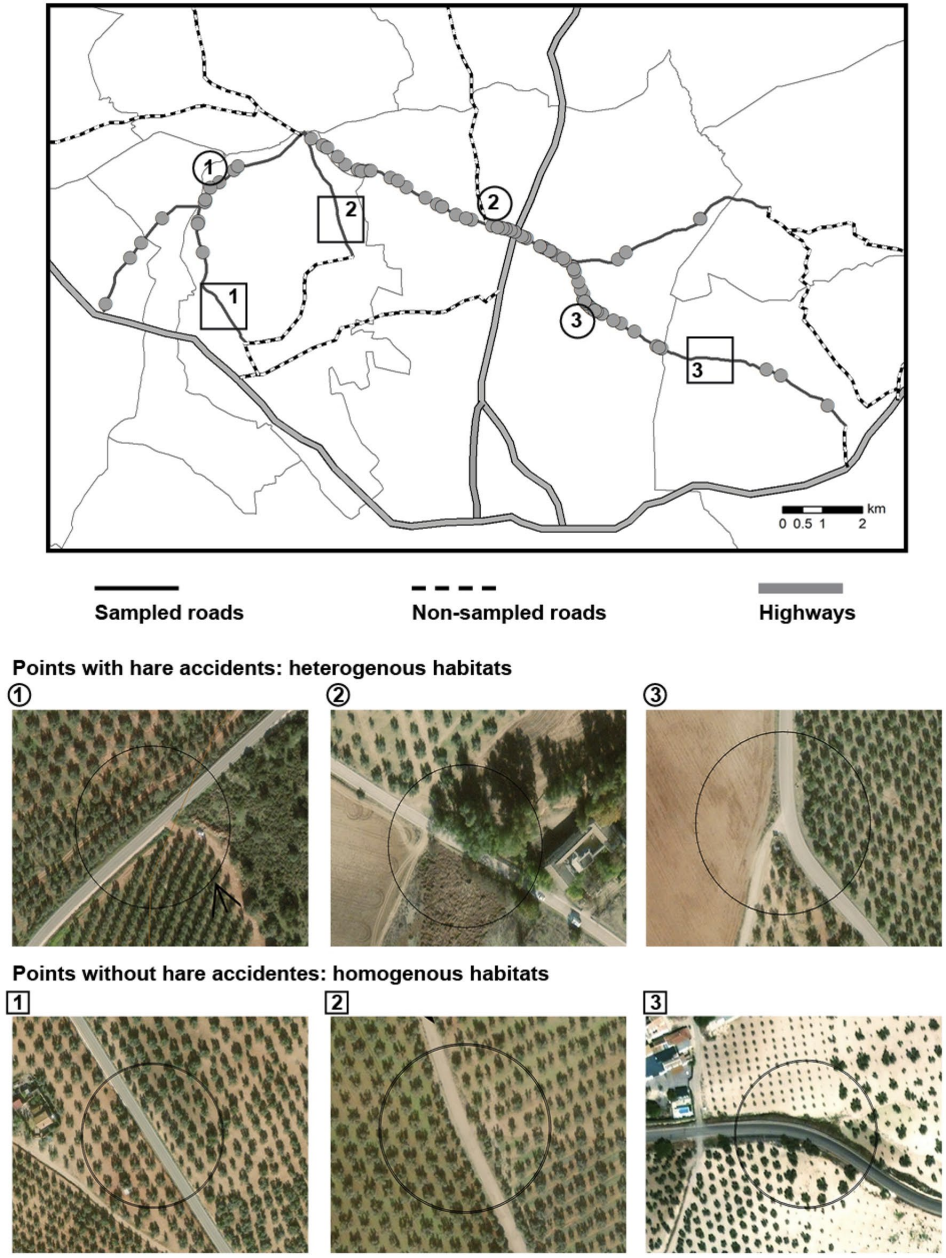
Representative points along the sampled road network with (heterogeneous habitat) and without (homogeneous habitat) hare accidents. Photographs show habitat within a 100 m buffer around each point; grey circles indicate points with hare roadkill events. Map created with ArcGis 9.3 (ESRI 2008). Datasets from Instituto de Estadística y Cartografía de Andalucía, Junta de Andalucía. Road network and administrative boundaries (https://www.ideandalucia.es/catalogo/inspire/srv/spa/catalog.search#/home) Ortophotography (https://www.juntadeandalucia.es/institutodeestadisticaycartografia/prodCartografia/ortofotografias/index.htm).

Hare roadkills were not randomly spatial distributed in either the 100 m road sections (Wald-Wolfowitz: N = 552; Z = − 5.782; p < 0.001) or the 500 m sections (Wald-Wolfowitz: N = 113; Z = − 4.024; p < 0.001), suggesting the possible existence of black spots. However, the variance/mean ratio (VMR) was > 1 only in the 500 m road sections (0.71 ± 0.12 hares killed per section; VMR = 1.21), confirming the existence of black spots in road sections of at least this size. A total of 68.7% of the hare accidents were concentrated in 18.8% (10.5 km) of the sampled road network (Fig. 3).

Throughout the entire length of the study period, the density of hares roadkills was 4.6 ± 0.5 hares/km^2^ in 100 m buffers and 0.9 ± 1.4 hares/km^2^ in 500 m buffers. The hunting yield in neighboring game estates was 15.1 ± 14.8 hares/km^2^. Therefore, roadkills are equivalent to 3–21% of the number of hares hunted in the area (Table 4).

**Table 4:** Mean monthly hare accident density in the study area (5 months of sampling) and ratio of hare accident density to monthly hare hunting yields (3 months of hunting season) in neighboring hunting estates (1993–2001 average).

	Hares/km^2^ per month (95% CI)	Roadkills/hunting yields (%) (95% CI)
100 m buffer (N = 552)	0.9 (0.7–1.1)	23.8 (22.3–24.9)
500 m buffer (N = 113)	0.2 (0.1–0.2)	4.7 (3.7–5.3)
Hunting yields (N_AHR_ = 485; N_GE_ = 71)	3.9 (3.2–4.5)	Not applicable

*N*_*AHR*_ number of annual hunting reports analyzed, *N*_*GE*_ number of game estates.

Tests for multicollinearity did not reveal any variables with VIF > 5. The best model (Table 5) correctly classified 92.5% of the accidents (n = 80) and 88.9% of the random points without collisions (n = 81). The accident points were associated primarily with landscape heterogeneity and secondly with road characteristics (embankments, crossroads, and traffic volume). A highly heterogeneous landscape, the presence of embankments and a nearby crossroad, and high traffic volume were the main factors influencing the occurrence of hare accidents (Table 6).

**Table 5:** Results of GLMMs explaining variation in hare–vehicle collision points and random points without collisions in the study area.

Model	k	AICc
Tf − Dc + Em + Lh	5	811.473
Tf − Dc + Em + Sl + Lh	6	817.349
Tf − Dc + Em + Sl + Lh	6	820.819
Tf − Dc + Em + Sl + Lh	6	868.006
Tf − Dc + Em + Sl + Lh	6	868.390
Tf − Dc + Em + Sl + Lh	6	907.247
Em + Lh	3	912.662
Tf − Dc + Em + Sl + Lh	6	924.308
− Dc + Em + Lh	4	1021.632
Null	1	1172.020

*Tf* traffic volume, *Dc* distance to nearest cross, *Em* presence of embankment, *Sl* speed limit, *Lh* landscape heterogeneity, *k* number of parameters, *AICc* Akaike information criterion corrected for small sample sizes.

**Table 6:** Results of the GLMM fitted to differentiate between hare–vehicle collision points and random points without collisions.

Source of variation	β ± SE	df	Wald	P
Landscape heterogeneity	1.547 ± 0.347	1	19.899	< 0.001
Presence of embankment	2.729 ± 0.616	1	19.661	< 0.001
Distance to nearest crossroad	− 0.207 ± 0.073	1	8.094	0.005
Traffic volume	2.789 ± 0.740	2	7.493	0.001

Model coefficients are shown with their standard error and Wald significance test. P values were considered to be significant at P < 0.05.

We detected an increase in traffic intensity on 46.3% of the surveyed roads between 2005 and 2017, showing that the number of vehicles/day has at least doubled^34,41^. The traffic in MA-6414 and MA-6415 increased from 1000 to 200 vehicles/day to 2000–5000. In MA-6409 traffic increased from 500 to 1000 to 1000–2000 vehicles/day. The rest of the roads kept the same traffic.

We estimated a mean rate of 5.8 ± 0.4 hare accidents per week (range: 4–8 hare roadkills) during the 2021 road surveys, which is similar to the rate observed in 2006. And updated hunting yields suggest an 87.6% decrease in the total number of hares captured for the province of Malaga from 2006 to 2019. However, the more detailed interviews with game managers, showed that the number of hares hunted had decreased by only 20% compared to 2006 in the study area.

## Discussion

The results of this study show that the Iberian hare is frequently involved in road accidents in the study area. We found that accidents were comprised in blackspots related to environmental and road features and a relevant level of road mortality that may represent up to 25% of the hunted hares in the area.

### Implications of road mortality for Iberian hare conservation

Our results indicate that over half of all documented vertebrate roadkills within the study area were Iberian hares. This frequency is higher than that observed for ungulates or other medium- and large-sized mammals^1,67^ and for other hare species^19,20^. In addition, the standardized kill rate was almost five times higher for Iberian hare than for the other studied hare species^17,18^, which contrasts sharply with other available data for hare road collisions in northern Spain^29^. We also found that almost two-thirds of the hare roadkills were concentrated in road black spots, as has been reported for other mammal species^13,68,69^. Given that mortality is concentrated in clearly defined road sections, roadkill mitigation measures should focus on these areas^70^.

However, it is unclear whether these results are representative of other areas in which the Iberian hare is present. Our conclusions are limited due to the short study period and a lack of replicates; therefore, our results should be considered preliminary with regards to the species’ road ecology. Further, smaller animals are readily missed during vehicle surveys, which could skew the frequency of hare roadkill detections^71^. Even so, we are confident in our findings suggesting that road mortality of hares is not insignificant and must be considered for successful conservation of the species. Two-lane roads comprise 89.4% of all Andalusian roads^35^; hence, the roads sampled in our study are representative of secondary roads throughout Andalusia. Fertile plains in Andalucía represent 31.1% of the regional landscape^72^, while olive groves, vineyards, and cereal fields cover 26.6% of the soil^30^. Therefore, the landscape conditions, soil use, and road network in the study area are typical of almost a third of the region.

Regarding the time elapsed since the initial surveys, the increase detected in traffic intensity may implies that if the densities of Iberian hares have remained the same in the study area over the past 15 years, the number of roadkill incidents should be similar to 2006 on roads where traffic intensity has not changed and should be higher on roads where the intensity has increased. With respect to hare populations, while hare accidents were similar to the initial survey, updated hunting yields^73^ suggest an important decrease in the total number of hares captured for the province of Malaga. However, we doubt that this result is representative of the study area; hares inhabit very different habitats throughout the province, many of them mountainous and unsuitable for the species, while the habitat in the study area is considered optimal for the species^23,40^. Interviews with game managers confirmed our doubts. Furthermore, one game manager pointed out that hare populations in the area suffered severe declines several years ago, from which they seem to have recovered only in the past 5 years. In summary, it appears that traffic intensity has increased on some roads in the study area but remained stable on others. Although hare populations have declined globally, local populations have recovered or remained stable, and the rate of hare accidents in the study area is similar after 15 years. Therefore, the results and recommendations from our analyses not only remain valid but are also increasingly relevant for hare conservation.

Another important limitation of the study arises from research published after our sampling period. It was shown that the carcasses of small mammals such as lagomorphs do not typically persist on roads for more than 1–2 days, particularly in summer due to the presence of scavengers, harsh weather conditions, or removal by people^74^. This suggests that, for a study such as ours, the optimal monitoring frequency for the detection of hare roadkills would have been daily rather than weekly. Further, the use of weekly rather than daily sampling could have resulted in a false negative rate in the estimated hotspots, missing “true” hotspots^75^. In the case of lagomorphs, we propose that weekly rather than daily sampling may have led to an underestimation of about 40%, which implies that our estimated roadkill mortality rate is much lower than the actual rate and some hotspots may have been missed. This supports the relevance of hare road mortality in the area and strengthens our results, as an underestimation of roadkill numbers implies a higher rate of additional mortality and increased bias in hunting quotas.

An additional consideration concerning the generality of our results is related to hare density. It has been argued that road mortality is not correlated with population densities and that traffic flow is the most important factor explaining variance in road accidents for certain taxa^76,77^. Although traffic does play an important role, a density-dependent relationship between roadkill numbers and population size has been demonstrated for wild rabbits^78,79^. This direct relationship suggests that similar processes may be operating for the Iberian hare. This relationship is true for hare hunting yields^80^ since higher hare densities and greater yields in fertile plains in which dry wood crops and irrigated herbaceous crops are intensively managed^23^ such as in our study area. Even considering the argument that traffic flow is more important than hare densities, the increase in traffic found on certain roads in the study area may be related to the stability of hare accident numbers despite the slight decrease in hunting yields (i.e., hare densities).

Finally, although our study period was short, it coincides with the period of maximum reproductive activity for this species. Phenology has been highlighted as the most important factor affecting temporal patterns in roadkill for small mammals^53^, and these patterns have been shown to repeat through time^74^ and ecoregions in a same region as Andalucía. Therefore, it is likely that our results may be repetitive in situations with similar habitat characteristics, road features, and hare populations, quantifying hitherto unknown rates of hare mortality and providing relevant information for species management^81^.

### Hunting and roadkill: a risk of additive mortality

Roadkill data have been used to improve species management planning both for endangered^82,83^ and game species^84–86^. Previous researchers have emphasized the general value of roadkill monitoring and its application to relevant ecological fields (e.g., as a source of information on population trends, patterns in species composition, or for mapping invasive species, contaminants, and diseases^81,87^). However, collision data for smaller game species are often neglected for these species’ management planning^79^.

In the case of hunting, the consideration of additional sources of mortality is fundamental, as their potential effects on species’ population dynamics^33,88^ could affect extraction rates^89^. Some authors consider vehicle collisions as an additive source of mortality^90,91^ and even a population sink^92^. Therefore, hares killed in collisions may represent individuals that would not have died if this cause of mortality did not exist. Moreover, hunting mortality is considered to be partially compensatory (i.e., individuals may have died due to other reasons if they were not hunted) whenever harvest rates are low^93^. However, at higher harvest rates, hunting mortality may also be additive. In such situations, harvest management should also consider unnatural sources of mortality and attempt to control these sources.

Iberian hare populations have changed significantly in the past 15 years. On a national scale, populations are thought to have decreased almost 49% from 2012 to the present, while hunting yields in the region of Andalusia have declined by 16%^94^. Under such conditions, even a low road mortality rate should be considered relevant and likely additive, especially when the species is also being threatened by new diseases^95,96^.

It has been argued that the high reproductive potential of Iberian hares facilitates population recruitment and could compensate for high hunting pressures even in cases of low hare density^27^. If this is the case, or if hare densities are high, the number of road-related fatalities could be insignificant. However, when populations are declining or being affected by diseases, road mortality should be considered since even a low number of accidents would have a clear density-dependent impact on populations. The combination of natural mortality, disease, and road collisions, as well as ineffective hunting plans, could drive populations to collapse. Regrettably, demographic compensation via increased fecundity of the remaining Iberian hare populations has not been well studied despite its potentially vital role in population growth and the prevalence of hare–vehicle accidents.

### Factors causing hare–vehicle collisions

As previous research has detected for other lagomorph species (e.g., *L. europaeus* in Brazil)^2^, landscape heterogeneity is the main factor influencing the prevalence of Iberian hare fatalities. Mixed patches of forest with pastureland or farmland create habitat mosaics where increases in resource availability for wildlife increase species abundances and therefore the likelihood of them crossing nearby roads^1,11^. The proximity of forests to open areas is also a key factor in collisions^97^. Road borders and verges may act as feeding areas for some species^14^ and also facilitate the movement of animals within their home range^98^.

Forested areas in our study region are comprised of groves of olive trees, a woody crop that provides water indirectly to smaller species through their widespread trickle irrigation systems in addition to providing food resources^99^. Weather conditions or seasonal variations affecting food availability may also influence roadkill rates^100^. This is likely to occur during the dry summers in the study area, which could push hares to cross roads in search of water. Similar to olive groves, vineyards also provide food, water, and refuge. It should be noted that Iberian hares follow a heterogeneous habitat selection pattern and move frequently between habitat patches^22^. Therefore, hares may be likely to cross roads at points of high landscape heterogeneity in search of food (i.e., herbaceous crop shoots, weeds, or early summer grapes), roadside vegetation, or road verges^8^; because of changes in food availability due to harvesting^101^; or in search of mates during the rooting season. This collection of factors influencing road collisions can appear to translate into a pattern of seemingly random accidents, as seen in this study, or accidents may simply occur frequently because hares cross the roads often.

The presence of embankments was the second factor favoring hare accidents. Previous studies have suggested that embankments act as barriers preventing animals from crossing the road^102,103^, and collisions occur when the road and the adjacent habitat are at the same level^104^. However, the difference in elevation on our sampled roads was never greater than 1 m and was less than 0.5 m in most cases. We do not believe that these slight differences prevent hares from accessing roads; however, may slow the hare’s ability to react when encountering a vehicle, increasing the likelihood of a collision.

In accordance with the findings of previous studies, most collision points were located near crossroads^2,3^. The effect of crossroads on road mortality differs between mammal species and may be related to the size of the animal involved. Ungulate collision points are usually far from crossroads^103^, which suggests that they may avoid these road sections or that large ungulates are easier to see and are therefore avoided by vehicles in these open areas. Gaps or discontinuations in roadside fencing at intersections may make medium- or large-sized mammals more prone to fatalities near crossings^51^. However, smaller species are less visible and hide in road verges^102,105^, making it more difficult to avoid collisions with these species near intersections. Small mammals can also dig or pass easily under the fences in the case of fenced roads. Finally, as previously established, higher traffic volumes are positively associated with wildlife fatalities^106^. This is one reason why some roads are fenced: to protect wildlife by preventing access to the roads. However, secondary and rural roads in Andalusia are rarely fenced.

### Possible roadkill mitigation strategies as conservation measures

Possible mitigation measures for Iberian hare accidents include improving habitat connectivity in the areas adjacent to roads, funneling animals towards crossing structures^107,108^, managing speed limits and traffic in sections with a high frequency of roadkill, fencing with adequate mesh sizes, or removing vegetation in road verges to create 50–100 m bands free of vegetation on both sides of the road^109^. However, most of these measures are costly to implement for existing roads, appear to do little to improve the situation^110,111^, require constant maintenance^112^, or may be contentious as, if inappropriately placed, fences could exacerbate barrier effects and have even greater negative impacts on the population than road mortality^113^. Further, individual variability in hares’ behavioral response to roads must be considered^52^; such research should be undertaken in black spots or carried out during seasonal peaks in road mortality^53^. Focusing mitigation efforts on black spots on new roads can be useful, but these sections may not be the best option for older roads due to population depression^114^.

Other optimal solutions must be sought^70^. In small or declining hare populations, roads may act as a clear threat and the creation of reserves without further fragmentation has been recommended^8^. The Andalusian network of protected areas is already quite extensive but does not include plains or cultivated lands, where the Iberian hare primarily resides. Hunting estates in the region may act as reserves for the protection of this species, and hunting also allows for the regulation of a species’ population ecology when harvesting is done sustainably. Given the economic and logistical obstacles that may arise in areas where most roads are old and go through private farmlands or hunting estates, we propose adjusting the hunting rates to compensate for the number of road accidents in the area as a first step in the mitigation of Iberian hare–vehicle accidents. This means that the number of hares killed on roads should be considered in hunting plans, without ignoring other mitigation measures. Therefore, we encourage careful consideration of traffic and road mortality when assessing the local population status of Iberian hares before devising hunting quotas. In addition, given that hare hunting may accrue economic benefits for the local estates, any proposed solutions in an interdisciplinary field such as road ecology must also consider whether reducing hunting quotas will have substantial monetary repercussions.

## Conclusions

Our results suggest future directions for theoretical and applied research in road ecology, including further exploration of the demographic compensation of roadkill and assessment of specific mitigation measures to protect species subject to exploitation, as is the case for the endemic Iberian hare. To compensate for mortality caused by road collisions, adjusting hunting quotes may be a simpler solution than habitat management to effectively contribute to the conservation of this species.
